# Changing the Landscape of Solid Tumor Therapy from Apoptosis-Promoting to Apoptosis-Inhibiting Strategies

**DOI:** 10.3390/cimb46060322

**Published:** 2024-05-28

**Authors:** Razmik Mirzayans

**Affiliations:** Department of Oncology, Cross Cancer Institute, University of Alberta, Edmonton, AB T6G 1Z2, Canada; razmik.mirzayans@ahs.ca

**Keywords:** solid tumor therapy, intratumor heterogeneity, polyploid giant cancer cells, senescence, anastasis, oncogenic caspases, Phoenix Rising, treacherous apoptosis, preclinical assays, precision oncology

## Abstract

The many limitations of implementing anticancer strategies under the term “precision oncology” have been extensively discussed. While some authors propose promising future directions, others are less optimistic and use phrases such as illusion, hype, and false hypotheses. The reality is revealed by practicing clinicians and cancer patients in various online publications, one of which has stated that “in the quest for the next cancer cure, few researchers bother to look back at the graveyard of failed medicines to figure out what went wrong”. The message is clear: Novel therapeutic strategies with catchy names (e.g., synthetic “lethality”) have not fulfilled their promises despite decades of extensive research and clinical trials. The main purpose of this review is to discuss key challenges in solid tumor therapy that surprisingly continue to be overlooked by the Nomenclature Committee on Cell Death (NCCD) and numerous other authors. These challenges include: The impact of chemotherapy-induced genome chaos (e.g., multinucleation) on resistance and relapse, oncogenic function of caspase 3, cancer cell anastasis (recovery from late stages of apoptosis), and pitfalls of ubiquitously used preclinical chemosensitivity assays (e.g., cell “viability” and tumor growth delay studies in live animals) that score such pro-survival responses as “lethal” events. The studies outlined herein underscore the need for new directions in the management of solid tumors.

## 1. Introduction

### 1.1. Expert Opinions on Solid Tumor Therapy

In 2016, Vinay Prasad published a Perspective article in Nature on the illusion of precision oncology, in which he concluded that “precision oncology is inspirational. What doctor or patient would not want to harness genetics to tailor a therapy to an individual? But traveling back in a time machine is also inspirational. Who would not want to wind back the clock to remove their cancer before it spreads? In both cases, however, as of 2016, the proposal is neither feasible, cost-effective nor assured of future success. Yet in only one of these cases does the rhetoric so far outpace the reality that we risk fooling even ourselves” [1].

In 2017, a Kaiser Health News article was published online by Liz Szabo, which contains input from oncologists and families of cancer patients regarding how “cancer treatment hype gives false hope to many patients” [2]. On the topic of being continuously bombarded with the news and scientific publications on how oncology is making significant progress and inroads in terms of winning the war against cancer, Dr. Otis Brawley, previous (2007–2018) chief medical officer at the American Cancer Society, stated that “We’re starting to believe our own (hogwash)… The consequences are real—and they can be deadly. Patients and their families have bought into treatments that either don’t work, cost a fortune or cause life-threatening side effects”.

Since then, numerous articles have been published in scientific journals (e.g., [3,4,5,6,7,8,9]) or posted online (e.g., [10,11,12,13,14]) that appear to support Prasad’s predictions. In 2023, for example, John Horgan [14] posted a blog in which he addressed “Big business, big hype (of cancer industry)…Little net progress besides anti-smoking efforts…The false comfort of survival rates…Corruption in the cancer industry…”.

### 1.2. Objectives

**Figure 1:**
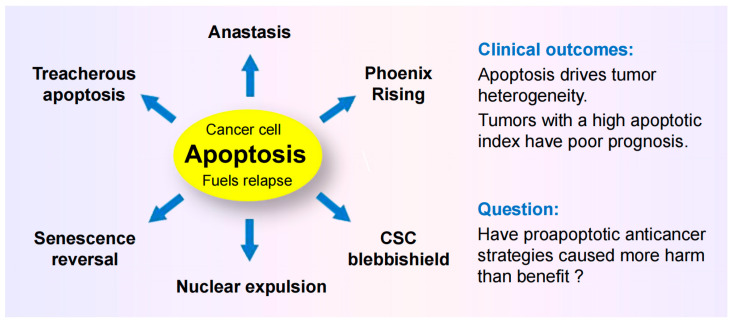
Dark side of apoptosis in treating solid tumor malignancies based on preclinical studies (**left**) and the outcome of clinical studies (**right**), which pose the indicated question.

The purpose of this review is to highlight several well-established therapy-induced responses that underlie resistance and relapse but surprisingly, continue to be widely overlooked. The graphic abstract regarding apoptosis is presented in Figure 1. The take-home messages are listed in Appendix A, which include an urgent need for a paradigm change to “stay away” from apoptosis-promoting strategies.

Specifically, this review is divided into four parts: (i) a brief discussion on the consequences of information-generating approaches (“omics”) to cancer therapy (Section 2); (ii) the contribution of genome chaos (e.g., polyploidy/multinucleation) to chemotherapy resistance (Section 3); (iii) the “dark” side of apoptosis in solid tumor therapy (Section 4); and (iv) exploiting the apoptotic threshold for managing solid tumors (Section 5).

## 2. Danger of Information-Generating Approaches to Cancer Biology: The Current State of Confusion

### 2.1. Off-Target Effects of Drugs Designed for Targeted Therapies

Over the past three decades, many cancer researchers and investigators in the pharmaceutical industry have expressed heightened excitement regarding the application of rapid screens and validation studies to identify drugs that are capable of targeting specific proteins. These so-called small-molecule inhibitors have been developed to be used for novel anticancer strategies under the term precision oncology with catchy names (e.g., targeted therapy, personalized medicine, synthetic “lethality”). Unfortunately, the majority (~97%) of such anticancer drugs that undergo clinical trials fail to advance to receive FDA approval [10,11,12,13,14,15,16,17,18]. For those few drugs that are approved, the results often turn out to be disappointing once they are administered in non-trial settings [13,14,15]. This is partly because of how trials are conducted, involving subjects who are “handpicked and generally in reasonable physical shape”, as practicing oncologist Azra Raza asserts in her book entitled “The First Cell: And the Costs of Pursuing Cancer to the Last” [15].

In 2019, Lin et al. [16] reported studies that were designed to investigate the target specificity of a set of cancer drugs that were in various stages of clinical trials. All drugs that were tested by these authors were shown to have off-target effects. In other words, many compounds that were identified/developed for “targeted therapies” and had entered clinical trials were not functioning for the purpose for which they were developed. This means that patients who participate in these clinical studies “risk their lives on treatments that end up in the dustbin” [12].

Since 2019, various groups have reported similar off-target effects for a number of other small-molecule inhibitors. These include poly (ADP-ribose) polymerase (PARP) inhibitors, which have been developed and evaluated in clinical trials for targeted therapies [19]. Unlike most of the drugs tested by Lin et al. [16], the PARP inhibitors olaparib, rucaparib, niraparib, and talazoparib have received FDA approval for various malignancies.

A summary of the results reported by Lin et al. [16] together with their clinical implications have been discussed in a blog by Julia Belluz [12]. The blog contains input from Nobel Prize Laureate William Kailen, who stated, “I hope this paper will help people see the need to raise to bar in terms of how we choose and validate cancer drug targets”. The blog also contains the following statement by Belluz: “In the quest for the next cancer cure, few researchers bother to look back at the graveyard of failed medicines to figure out what went wrong” [12]. (Our group has been in the same boat, as stated previously [20,21]).

This is one of the main goals of the proceeding discussions. Namely, to try to shed some light on what went wrong in the “graveyard” of precision oncology.

### 2.2. Precision Oncology for Treating Patients with Solid Tumors: Ever-Increasing Information and More (Empty) Promises

A decade ago, Robert Weinberg published a leading-edge essay article entitled “Coming Full Circle—From Endless Complexity to Simplicity and Back Again”, in which he discussed the consequences of a merely reductionist approach to cancer research. The reductionist paradigm refers to the hypothesis that biological systems can be fruitfully investigated at the lowest possible level [22]. In this essay, Weinberg noted that, since the 1980s, the need for generating large data sets led to the “omics” era of cancer research, which included studying genomes, transcriptomes, proteomes, epigenomes, kinomes, methylomes, glycomes, and matrisomes. Each one of these “omics” branches led to the accumulation of staggering amounts of information. Unfortunately, however, we can’t really assimilate and interpret most of the accumulated data [22]. More recently, William Kailen also discussed the danger of relying on large amounts of data sets, often crammed into a single manuscript, without undertaking in-depth biological assessments to validate overly broad claims in terms of potential clinical relevance [11,23]. Kaelin used the analogy of “building with straw” for such information-generating publications and stated that “real advances are built with bricks, not straw” [23]. Kaelin also cautioned the scientific community in terms of drawing “wishy-washy” conclusions from the ubiquitous use of “down” assays (see below) to clinical relevance. (Key points raised by Weinberg, Kailen, and other leading oncologists in the context of solid tumor therapy have been recently reviewed [24]).

**Figure 2:**
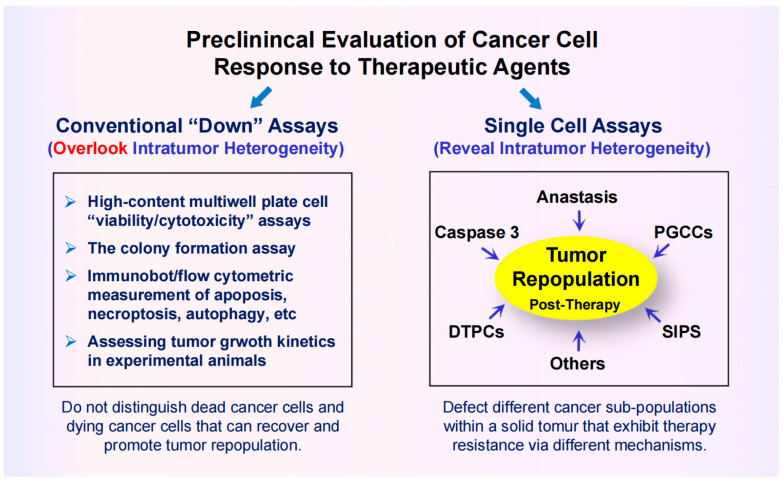
Preclinical “down” assays that are widely used to identify novel anticancer drugs and treatment strategies (**left**) measure averaged responses of a large number of cells. Such assays are not designed to recapitulate the complexity and heterogeneity that exists within a tumor in terms of therapy resistance (**right**).

The commonly used “down” assays include immunoblotting (to assess decreased global expression of genes/proteins under study), decreased cell proliferation, and decreased tumor growth in live animals [11,23]. A major shortcoming of such assays is that they overlook the complexity and heterogeneity that exists within a tumor (intratumor heterogeneity) ([24,25]; also see Figure 2). Another shortcoming pertains to experimental design in most apoptosis and cell “viability” studies (see Section 5 below).

### 2.3. Anticancer Strategies Targeting the p53 “Firework”

In 2017, Kastenhuber and Lowe [26] discussed the difficulties associated with targeting the p53 network in the context of cancer therapy and concluded that, despite over three decades of intensive research and numerous discoveries, “a clear appreciation of how and in what contexts p53 exerts its diverse effects remains unclear”. Since then, many other authors have discussed the increasing challenges encountered in p53/p21-based cancer therapeutic strategies (too many to cite!). These challenges in part pertain to proteoforms of p53 (and its family members, p63 and p73) [26,27,28,29,30], which can be generated by posttranslational modifications, alternative splicing, mutations, and a combination of these events [27,30]. Uveresky [27] has appropriately used the term “p53 firework” (rather than network) to illustrate the mind-numbing complexity of p53 proteoforms.

The firework extends to p53 downstream events, given its well-known function as a transcription regulator. A meta-analysis reported in 2017 identified over 3500 genes that are directly upregulated by p53 [31]. In addition, p53 interacts with a large number of proteins [32] and contributes to the stability of the epigenetic state [33,34].

**Figure 3:**
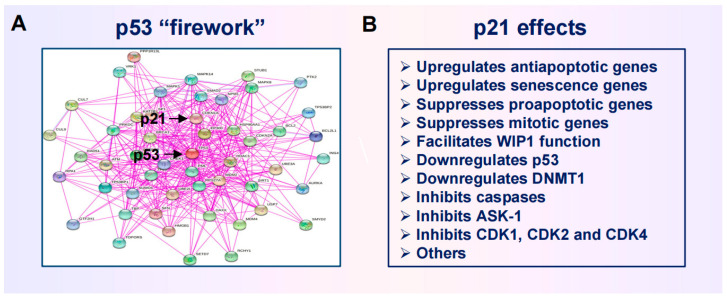
(**A**) Some p53-interacting proteins that create the p53 “firework” (free online available information presented in [32]). (**B**) Multiple functions of p21. CDK, cyclin-dependent kinase; ASK, apoptosis signal-regulating kinase; DNMT1. DNA methyltransferase 1.

An illustration of p53-interacting proteins is available online (cited in [32]). Figure 3 contains this version of “p53 firework” together with key functions of the p53 target p21^WAF1^ (p21). Like p53, p21 regulates gene expression both directly and indirectly by modulating the epigenetic landscape. (The multifunctional nature of p21 beyond its canonical role in regulating cell cycle progression has been reviewed [35]). 

Many efforts have been devoted to predicting the biological outputs of the molecule(s) under study merely based on such perplexing signaling networks. As an example, Markowska et al. [36] have recently presented a “firework” of overlapping signaling pathways that regulate chromatin remodeling, DNA damage response, and cell cycle. The authors used a uniquely comprehensive workflow and identified several pairs of genes, related to these processes, that might display synthetic “lethal” interactions. (This phenomenon refers to genetic interactions between two genes, where inactivation of each individual gene is compatible with a viable phenotype, whereas co-inactivation of both genes is anticipated to result in lethality [36]).

The danger of relying on such an information-generating approach for predicting potential cell fate outcomes is that key discoveries of fundamental clinical relevance might be overlooked. For example, in most reviews on different strategies of precision oncology (e.g., engaging apoptosis [37,38,39,40,41,42], targeting the p53 pathway [43,44,45,46,47], synthetic “lethality” [48,49,50,51,52,53,54]), there is no mention of “outliers” within a tumor that are known to underlie resistance and relapse. These outliers include cancer cells with various manifestations of genome chaos and apoptotic cancer cells that survive and generate aggressive variants (see Section 3 and Section 4).

With respect to synthetic “lethality” targeting the DNA damage response, several challenges in implementing this strategy were discussed by Lord and associates in a landmark review that was published in 2017 [53]. Since then, the challenges have mounted, which led Groelly et al. [49] to caution that such an approach might not work for complex diseases such as cancer. 

*Please note:* The current review is focused on solid tumors, and thus the conclusions drawn may or may not pertain to other malignancies. In addition, quotation marks are used for the term “lethality” throughout this review, given that in vitro colony formation and other preclinical assays that are widely used to assess “lethality” are not designed to distinguish between dead cancer cells and dying cancer cells that can recover and contribute to disease relapse. 

## 3. Impact of Genome Chaos on Cancer Cell Resistance to Therapeutic Agents

One of the reasons why the metaphoric “war on cancer” has not been won is the ability of cancer cells to evolve and undergo phenotypic changes (plasticity). Ironically, these adaptation processes are greatly enhanced by our anticancer treatment strategies. In other words, the various cytotoxic treatment options that are designed to destroy cancer cells (e.g., via regulated cell death) can paradoxically trigger the creation of more aggressive “monsters” [55,56,57,58,59]. This rather bizarre process of acquired therapy resistance of cancer cells is characterized by rapid and extensive genome restructuring episodes (i.e., through non-mutational/non-genetic events) and has been termed “genome chaos” by Heng and coworkers ([56,57]). 

### 3.1. Therapy-Induced Dormancy via Polyploidy/Multinucleation

**Figure 4:**
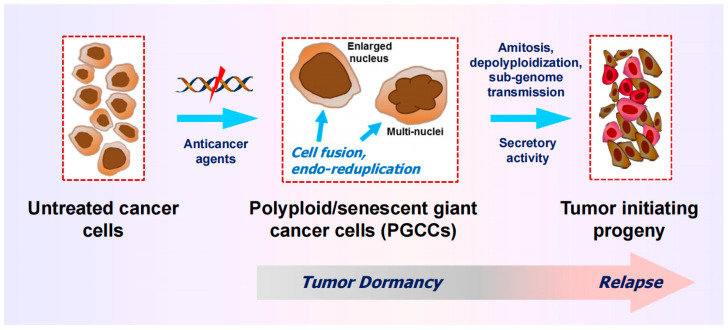
Cartoon illustrating the development and fate of polyploid giant cancer cells (PGCCs), containing a highly enlarged nucleus or multiple nuclei. PGCCS often enter a state of dormancy after they are formed but remain viable and secrete growth-promoting factors. PGCCs can also give rise to therapy-resistant and tumor-repopulating progeny through amitotic cell division, depolyploidization, and sub-genome transmission of nuclear material into surrounding cells. For further details, see [25].

A subset of cancer cells with a chaotic genome are readily distinguishable from the bulk of cancer cells by virtue of their enormous size and massive nuclear contents (i.e., a highly enlarged nucleus, multiple nuclei, and/or multiple micronuclei). These so-called polyploid giant cancer cells (PGCCs) can enter a state of transient dormancy (active sleep) after they are formed, but remain viable, secrete growth promoting factors, and exhibit the ability to undergo depolyploidization as well as “neosis” (nuclear budding and bursting), ultimately giving rise to stem-cell-like progeny that repopulate the tumor (reviewed in [25]; also see Figure 4). 

The mechanisms of formation and fate of PGCCs as well as their prevalence and prognostic value across different cancer types are well established and extensively discussed, albeit by only a handful of authors who reply on time lapse microscopy and other single cell assays (e.g., [59,60,61,62,63,64,65,66,67]).

### 3.2. The Creation of PGCCs Complicates the Interpretation of Chemosensitivity Data

PGCCs are not considered by most authors who have discussed the challenges and promises of precision oncology, synthetic “lethality”, artificial intelligence, and p53/p21-based therapies (too many to cite!). Possible reasons for this serious oversight have been pointed out [24,25], which include misinformation perpetuated by the misguided use of preclinical chemosensitivity studies (e.g., the colony formation assay, multi-well plate “viability” assays, and tumor growth studies in live animals).

**Figure 5:**
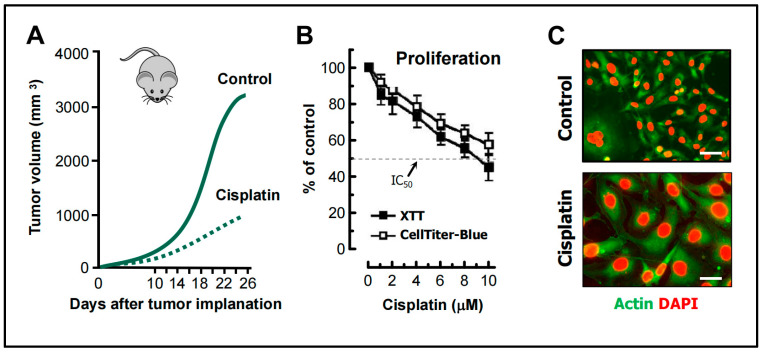
(**A**) The influence of cisplatin treatment on tumor growth in live animals (redrawn from [68]). (**B**) Sensitivity of MDA-MB-231 breast carcinoma cells to cisplatin (3 days continuous treatment) as measured by the indicated 96-well plate cell proliferation assays. (**C**) Fluorescence images showing the morphology of MDA-MB-231 cells before (control) and after treatment with cisplatin (10 µM; 3 days). Scale bars, 30 µm. Reproduced from open access publications [25,68,69].

The in vivo and in vitro cisplatin-triggered responses presented below illustrate the consequences of relying on such assays. The tumor growth data shown in Figure 5 are reproduced from a study reported by Weng et al. [68], and the cell-based data are reproduced from our published work [69]. The following observations should be noted:

- Weng et al. [68] studied the mechanism of cisplatin resistance in B16-F10 (mouse melanoma) cells grown in C57BL (immune-proficient) mice. As expected, cisplatin treatment resulted in a significant reduction (by ~75%) of tumor volume compared to the control group (Figure 5A). Tumors in cisplatin-treated mice were highly enriched with PGCCs, which were shown to be more malignant than the parental cancer cells. A similar observation was reported by Puig et al. [70] with rat colon carcinoma isografts established in immunocompetent rats. In the latter study, tumor repopulation resulting from PGCCs was observed late (>40 days) after cisplatin treatment (for details, see [25]).
- Figure 5B shows the cisplatin sensitivity of MDA-MB-231 breast carcinoma cells evaluated by the 96-well plate XTT and CellTiter-Blue assays. The IC_50_ (half-maximum inhibitory concentration) values were ~10 µM.
- Further studies involving single cell assays demonstrated that cisplatin sensitivity reflected proliferation arrest via the creation of PGCCs (predominantly mononucleated giants; Figure 5C) and that virtually all emerging PGCCs remained adherent to the culture dish and remained viable and metabolically active [69].

Data interpretation: The data obtained by such “down” assays (Figure 5A,B) are typically misinterpreted to represent cancer cell death. However, based on single cell evaluations (Figure 5C and [68,69,70]), as we stated previously ([20,25]), these chemosensitivity assays measure the conversion of dangerous (proliferating) cancer cells to potentially even more dangerous (proliferation arrested) “monsters” (e.g., PGCCs) that are known to repopulate the tumor. 

### 3.3. Common Features of PGCCs and Senescent Cancer Cells

Therapy-induced dormancy in p53 wild-type cancer cells predominantly reflects p21-mediated senescence. Landmark studies reported from the laboratory of Igor Robinson over two decades ago demonstrated that p21 orchestrates this response by transcriptionally upregulating senescence genes and down regulating genes involved in mitosis (reviewed in [71]).

It was originally assumed by us [72] and many other groups that therapy-induced senescence might be a permanent cell fate (i.e., reflecting “reproductive” death [73]). Now we know that: (i) senescence can be reversible, particularly in the absence of p16^INK4A^ (p16) function [71,74]; (ii) engaging regulated cell death via apoptosis in senescent cancer cells (e.g., MCF7 breast carcinoma cells that lack p16 function) can accelerate the reversal of senescence rather than leading to lethality [75]; (iii) senescent cancer cells that emerge following chemotherapy exposure gain survival advantage by frequently engulfing neighboring (senescent or non-senescent) tumor cells [76]; and (iv) senescence can be accompanied by polyploidy/multinucleation and the acquisition of several stem-cell-like characteristics [70,77,78].

In the context of cancer therapy, giant cancer cells with a highly enlarged nucleus (e.g., Figure 5C), multiple nuclei, and multiple micronuclei, with or without senescence features, together with senescence cells with modest (if any) nuclear abnormalities, exhibit the following common properties: they enter a state of dormancy (and thus might be scored “dead” in various chemosensitivity assays), remain viable, secrete growth promoting factors, and can give rise to rapidly proliferating progeny with stem-cell-like properties (reviewed in, e.g., [25]).

## 4. Rethinking Pro-Apoptotic Strategies to Combat Solid Tumors

Apoptosis is the most extensively studied form of regulated cell death and has been traditionally considered to be irreversible once a particular biochemical point-of-no-return is activated. For educational purposes, Taylor et al. [79] used the analogy of demolishing a building to illustrate the complex process of apoptosis. The demolition of a building needs to be carried out in a safe, precise, and orderly manner to avoid damage to surrounding buildings, and the resulting debris also needs to be removed to make room to build a new structure. Previously, it was assumed that cell demolition via apoptosis followed a similar scenario [79]. In the past 15 years, however, numerous groups have demonstrated that apoptosis is a reversible process and that triggering cancer cell apoptosis can fuel tumorigenesis. 

The processes of cell survival after engaging apoptosis and other modes of regulated cell death are well established and extensively reviewed (e.g., [80,81,82,83,84,85,86,87,88,89,90,91,92,93]). Some key discoveries are outlined below to illustrate the basis for the main purpose of the current review: a paradigm shift in oncology to focus on apoptosis-suppressing (rather than promoting) strategies.

### 4.1. Dark Side of Apoptosis in Solid Tumor Therapy

#### 4.1.1. Pro-Survival Function of Caspase 3

Caspase 3, which has canonically served as a marker of programmed cell death, is known to promote survival through processes that are called “Phoenix Rising” [82,88,89] or “Failed Apoptosis” [80,83,86]. These processes are initiated by DNA double-strand breaks (e.g., arising in the course of regulated cell death), resulting in the activation of stress response signaling pathways (e.g., orchestrated by ATM) and caspase 3-mediated secretion of prostaglandine E2 and other prosurvival factors. (This calls for revisiting thousands of articles that have relied on caspase 3 activation as a marker of cell “lethality”).

#### 4.1.2. Anastasis

The term “anastasis” refers to a homeostatic process that enables mammalian cells to recover after engaging regulated cell death [81,84,85,87,94,95]]. Like the prosurvival function of caspase 3, anastasis poses an obvious challenge in cancer therapy. Thus, cancer cells triggered to undergo apoptosis (e.g., in response to chemotherapeutic drugs) can recover from the late stages of apoptosis (even after the formation of apoptotic bodies) [91,94] and give rise to aggressive variants with increased aneuploidy [94,95]. (This calls for revisiting thousands of articles that use the terms “apoptosis” and “lethality” interchangeably!).

#### 4.1.3. Treacherous Apoptosis

Studies with adenocarcinoma tumor samples have revealed the presence of densely populated apoptotic cells within an individual tumor [96]. Such “apoptotic islands” contribute to intratumor heterogeneity and are associated with cancer cell survival [96]. This process of cancer cell survival—called “Treacherous Apoptosis” by Dhanasekaran [90]—cannot be recapitulated by the conventional preclinical “down” assays listed in Figure 1.

#### 4.1.4. Nuclear Expulsion

In a recent report, Park et al. [97] demonstrated that apoptotic cancer cells can promote metastasis through a process called nuclear expulsion. This involves the release of chromatin and associated proteins (nuclear expulsion products; NEPs) by apoptotic cells, which bind to neighboring cancer cells and lead to their metastatic outgrowth in an ERK-dependent manner. NEPs were detected in cancer cell lines as well as in tumor biopsies from patients with different types of solid tumors. The authors concluded that apoptosis-induced nuclear expulsion might be a generalized phenotype in cancer that underlies metastatic spread. The relationship (if any) between this process and treacherous apoptosis remains to be determined.

#### 4.1.5. Relationship between Apoptosis and Cancer Aggressiveness

Clinical studies reported since the 1990s have revealed that solid tumors with a high apoptotic index tend to have a poor prognosis. The dark side of apoptosis based on clinical outcomes was well documented over a decade ago [98,99,100] and has been fairly recently reviewed for colon cancer [101,102] and breast cancer [103,104,105].

### 4.2. Cancer Stem Cell Survival after Engaging Apoptosis

Since anastasis is a homeostasis cell recovery process, it is likely to occur in all cell types, including stem cells. In 2016, Jinesh and coworkers reported an anastasis-like phenomenon of survival in cancer stem cells after triggering apoptosis [106]. One of the typical morphological changes of apoptotic cells is the formation of membrane blebs, which are initially small, but their size gradually increases during the progression of apoptosis, eventually leading to the formation of apoptotic bodies (large extracellular vesicles), that are subsequently phagocytosed [107]. Apoptotic cancer stem cells were shown to avoid death (phagocytosis) by fusing their blebs to form blebbishield. This intriguing phenomenon (blebbishield emergency program) will undoubtedly attract more attention, and its relation to anastasis will be determined.

### 4.3. Questions

Collectively, these observations raise two fundamental questions. First, do TUNEL (terminal deoxynucleotidyl transferase dUTP nick end labeling) staining, Annexin V staining, and other apoptosis assays measure cancer cell death in preclinical studies? Second, why are novel anticancer strategies centered on promoting cancer cell apoptosis rather than avoiding/suppressing this “treacherous” response? Perhaps because many authors still consider apoptosis to be a permanent cell fate (e.g., [37,38,39,40,41,42,43,44,45,46]).

## 5. Exploiting the Apoptotic Threshold for Managing Solid Tumors

### 5.1. Apoptosis-Promoting Preclinical Chemosensitivity Studies Often Generate Clinically Irrelevant Information

**Figure 6:**
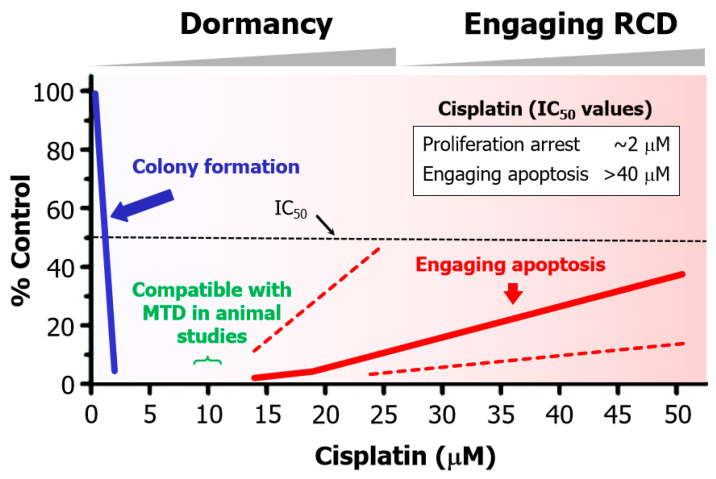
Apoptotic threshold in human HCT116 colon carcinoma cells treated with the chemotherapeutic drug cisplatin. The apoptotic trend shown by the sold red curve is based on caspase 3 activation reported by Berndtsson et al. [109] in 2006. Since then, other groups have used different assays and confirmed that very high concentrations of cisplatin are required to engage apoptosis in a significant proportion of cells within HCT116 cultures. For example, 60 µM cisplatin induced ~20% apoptosis [111], and ~100 µM cisplatin induced ~30% [112] and ~60% [113] apoptosis in this cell line. In our own work [110], we observed higher levels of apoptosis compared to other groups, but, consistent with other groups, we also observed marginal levels of apoptosis at the MTD (~10 µM). These various apoptotic responses reported from different laboratories are marked with two red dashed lines. MTD, maximum tolerated dose.

Figure 6 presents a summary of cisplatin-induced responses in human solid tumor-derived cell lines that were reported over a decade ago (reviewed in [108]). The following observations should be noted:

- Cisplatin concentrations between 5 and 10 µM used in cell-based studies are determined to be comparable to concentrations that may be achieved in tumor/tissues of treated patients and tumor-bearing laboratory animals [70]. Higher concentrations result in severe side effects in animal studies [70]. Thus, ~10 µM cisplatin is denoted as the maximum tolerated dose (MTD) in Figure 6.
- The IC_50_ (half-maximum inhibitory concentration) values for inhibition of cell proliferation are ~2 µM when determined by the colony formation assay [109] or direct cell counting [110].
- The IC_50_ values for engaging apoptosis are way above the MTD [109,110,111,112].
- The basis for this discrepancy is known [109]. Relatively low concentrations of cisplatin induce sufficient amounts of DNA lesions that inhibit cell proliferation, whereas very high drug concentrations are needed to damage the cytoplasmic compartments to engage apoptosis.
- Such a discrepancy in IC_50_ values for inhibition of cell proliferation and induction of apoptosis has been observed for various solid tumor-derived cell lines after exposure to cisplatin or other chemotherapeutic drugs (e.g., oxaliplatin, paclitaxel, and doxorubicin) (for details, see [108,109,110]).

In short, cell “viability” (e.g., 96-well plate colorimetric) and apoptosis studies that call for continuous treatments with very high concentrations of chemotherapeutic drugs (e.g., >10 µM cisplatin) to achieve the IC_50_ values have probably generated clinically irrelevant information. The danger of relying on such assays for chemosensitivity assessment has been discussed by us [20,24,84] and others [85,114,115]

### 5.2. Chemotherapy-Induced Cancer Cell Dormancy: Lesser Evil Than Apoptosis?

The difference in drug concentrations that are required to induce proliferation arrest versus apoptosis is striking. We have presented the trend for cisplatin (Figure 6) and oxaliplatin [116], but similar differences are reported for virtually all anticancer agents. These observations led us to suggest that, in the absence of a “magic bullet” for treating cancer, perhaps cancer cell dormancy might be a more favorable clinical outcome than apoptosis simply because relatively low/moderate doses of therapeutic agents that are required to trigger dormancy “will undoubtedly cause less unwanted side effects, such as compromising the patient’s immune system, as compared to high doses required to induce apoptosis” [117].

## 6. Conclusions

### 6.1. Preclinical “Down” Assays have Caused More Harm Than Benefit

For nearly half a century, most cancer researchers and oncologists have relied on the ubiquitous use of “down” assays (e.g., decreased proliferation, decreased tumor growth, etc.) in concert with omics technologies to draw conclusions of clinical relevance. Despite generating huge amounts of bewildering information, modern therapeutic strategies under the term “precision oncology” have not fulfilled their promises for treating patients with solid tumors.

Such information-generating strategies, which include targeting p53, are largely centered on the hypothesis that engaging apoptosis in cancer cells will eventually lead to their demise. This is surprising because clinical studies published since the 1990s have revealed that this rather simplistic hypothesis is not tenable. Consistent with these clinical observations, in the past decade numerous authors have demonstrated that cancer cells undergoing apoptosis not only secrete pro-survival factors, which are mediated by caspase 3, but also exhibit the ability to recover from late stages of apoptosis (even after the formation of apoptotic bodies) via the homeostatic process of anastasis.

A paradigm shift is urgently needed in terms of how potential anticancer drugs are identified and validated (i.e., by single-cell techniques and not conventional “down” assays), as well as the rationale for this endeavor (i.e., suppressing, rather than promoting regulated cell death). Exploiting the apoptotic threshold (Figure 6) and the antiapoptotic property of wild-type p53 signaling [25,118] might prove to be instrumental in this regard.

This paradigm shift needs to encompass cancer cells with chaotic genomes in general, and PGCCs in particular. To this end, it is curious that these tumor repopulating giants still continue to be ignored by the majority of precision oncology community members. “Are misleading/inappropriate preclinical assays to be blamed” [24]? If so, then it is reasonable to conclude that widely used preclinical “dawn” assays have derailed cancer research for decades.

### 6.2. Conflicting Reports on Cancer Cell Fate after Engaging Apoptosis

Comprehensive review articles have been published by two members of the Nomenclature Committee on Cell Death (NCCD), Douglas Green and associates [92] and Boris Zhivotovsky and associates [85], as well as other authors (e.g., [93]), in which they have extensively discussed the mechanisms of cancer cell survival after engaging regulated cell death. These articles have concluded that “…the goal of most cancer therapies (to induce regulated cell death in cancer cells) directly brings about the cellular changes that we seek to avoid” [92], “…the identification of the anastasis phenomenon and the accumulated data on it indicate the importance of developing a new direction in the study of tumor resistance” [85], and “…while we know that cells respond differently to caspase activation, we still don’t fully understand where this heterogeneity comes from…what is the point of no return in apoptotic commitment?” [93].

On the other hand, a number of other comprehensive reviews on apoptosis in cancer [37,38,39,40,41,42] and related topics (e.g., p53-based therapies [43,44,45,46,47], synthetic “lethality” [48,49,50,51,52,53,54]) have been recently published in high impact factor journals that completely ignore the dark side of apoptosis in cancer therapy. These include a recent Nature review article published by the NCCD, which states (under the FACTS) that “In mammalian organisms, executioner caspases are activated after cells are already committed to die” [37]. What is the reason that the NCCD members, which include over 300 world-class experts, have provided an impressive account of apoptotic pathways in relation to cancer (and other diseases) in a literal “dictionary-style” article but have left out the fundamental responses listed in Figure 1 (anastasis, Phoenix Rising, etc.) that are known to underlie cancer resistance and disease recurrence? “I wouldn’t pretend to know”. (This quote is from Weinberg’s 2014 Leading Edge Essay [22], when he was discussing how this data-generating dilemma of cancer research will play out.).

### 6.3. Intratumor Heterogeneity: How Complex Does It Get?

Therapy-resistance responses of cancer cells are not limited to those discussed in the previous sections. The immune system, for example, plays an important role in therapy outcomes [24], which underscores the limitations of preclinical anticancer studies that focus on immune-compromised animals (e.g., nude mice). Other therapy-resistance responses that contribute to intratumor heterogeneity have been discussed. These include: (i) atavistic reprogramming, which refers to the activation of ancestral resistance genes [24], some of which might contribute to cancer cell escape from immune editing; (ii) cell fusion, which is known to promote the creation of PGCCs and facilitate their migration [20]; (iii) cancer cell dormancy reflecting drug-tolerant persister cells [20]; and (iv) the ability of p21 to promote cancer cell survival following clinically relevant chemotherapy exposure through a process called the “p21 Goldilocks Zone for Proliferation” [119].

### 6.4. Effective Anticancer Strategies Need to Target More Than One Therapy-Resistant Cancer Sub-Population within a Tumor

There is a common trend in most articles that discuss the significance of the specific therapy resistance response under study (e.g., anastasis, oncogenic caspase 3, PGCCs, DTPCs, SIPS, and others; see Figure 2, right panel). Most articles reporting the dark side of apoptosis, for example, rush to conclusions in terms of clinical relevance (e.g., “targeting anastasis during chemotherapy may prevent tumor relapse and metastasis”) without considering other therapy resistance responses. Most articles on drug-tolerant persister cancer cells rush to conclusions (e.g., “Our study…establishes a novel therapeutic strategy for…preventing tumor recurrence”) but totally disregard the numerous other resistance responses reported by other authors. Similarly, most articles on targeting p53/p21 that propose promising future directions disregard other aspects of therapy resistance, and so on.

A very disturbing picture emerges when considering the fact that all of these various resistance responses can occur in different sub-populations of cancer cells within an individual tumor. Accordingly, an effective therapeutic strategy needs to target more than one aspect of intratumor heterogeneity.

### 6.5. Where Will All This Lead?

Just thinking about all these complex processes makes one’s head spin, but unfortunately this is the established reality. We will be fooling ourselves if we continue to “reduce” this mind-numbing complexity to highly simplistic hypotheses, such as the notion that cancer cells treated with therapeutic agents either repair their genome and survive or die via apoptosis and other means.

Time will tell whether there will be a significant improvement in solid tumor therapy or whether precision oncology will remain an illusion as predicted by Prasad a few years ago [1]. Until then, hopefully taking heed of the advice of numerous pioneers in cancer research, including those noted herein, will prevent the undertaking of unnecessary clinical trials to test flawed strategies based on faulty hypotheses. 

### 6.6. Further Reading

This review was not intended to provide an in-depth discussion of all aspects of therapy resistance in different types of malignancies. For those who are interested in further reading, we suggest recent reviews by our group and others on related topics such as the dark side of regulated cell death in solid tumor therapy [20,33,84,85,92], cancer cell dormancy and disease recurrence post-therapy [20,59,62], the challenges and promises of cancer immunotherapy [120,121], the significance of wild-type p53 signaling in suppressing (treacherous) apoptosis [21,118,122], the impact of host immune response on the outcome of cancer therapy [24,123], the role of tumor microenvironment in therapy resistance and disease relapse [124], and tumor cell plasticity [125,126].

It is also important to note that some of the caveats regarding progress in cancer research pertain to publications in major journals that contain massaged or falsified results (see, e.g., Section 4.3 in [25]). The consequences of dishonesty in data reporting have been discussed in various articles (reviewed in, e.g., [24,25]), including a recent blog by Kelsey Piper [127] entitled “Who Fakes Cancer Research? Apparently, Lots of People” [127]. In short, faked data is the latest sign of a fraud problem in cancer research [127,128].

## Figures and Tables

**Figure A1:**
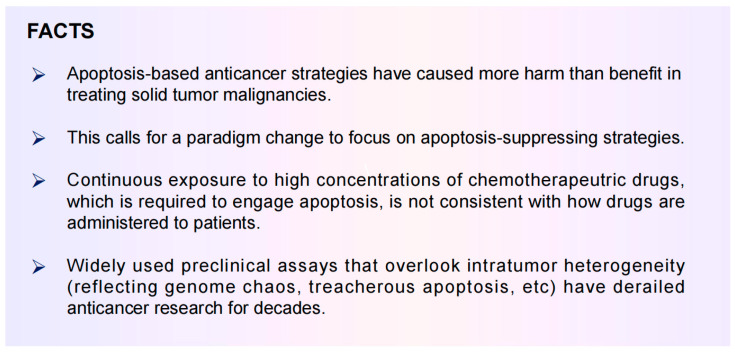
Take-home messages (FACTS) of the studies discussed in the current review.
